# The Effect of Perioperative Topical Ketorolac 0.5% on Macular Thickness after Uneventful Phacoemulsification

**DOI:** 10.1155/2017/4271671

**Published:** 2017-11-23

**Authors:** Derya Dal, Ozge Sarac, Yasin Toklu, Ayse Gul Kocak Altintas, Hasan Basri Cakmak, Elif Damar Gungor, Saban Simsek

**Affiliations:** ^1^Department of Ophthalmology, Erzurum Regional Training and Research Hospital, Erzurum, Turkey; ^2^Department of Ophthalmology, Ankara Ataturk Training and Research Hospital, Faculty of Medicine, Yildirim Beyazit University, Ankara, Turkey; ^3^Department of Ophthalmology, Ulucanlar Eye Training and Research Hospital, Ankara, Turkey; ^4^Department of Ophthalmology, Faculty of Medicine, Hacettepe University, Ankara, Turkey; ^5^Department of Ophthalmology, Malatya Training and Research Hospital, Malatya, Turkey

## Abstract

**Background:**

To evaluate the effects of topical 0.5% ketorolac treatment combined with topical steroids on macular thickness in cases who had uneventful phacoemulsification surgery.

**Methods:**

58 eyes of 58 consecutive cases were included. The mean foveal thickness (MFT), parafoveal thickness (ParaFT), and perifoveal thickness (PeriFT) measurements were performed with optical coherence tomography (RTVue-100, Optovue, Fremont, CA, USA) preoperatively and at postoperative 1 week, 1 month, and 2 months. All cases received topical 0.1% dexamethasone postoperatively. Randomly selected cases additionally received topical 0.5% ketorolac, which started 2 days prior to surgery. Cases who received both topical steroids and ketorolac formed group 1 and subjects who received only topical steroids formed group 2.

**Results:**

The increase in mean MFT at the 1st week, 1st month, and 2nd months after surgery in group 1 was significantly lower than group 2 (*P* = 0.008, *P* ≤ 0.001, and *P* ≤ 0.001, resp.). In group 1, the increase in mean ParaFT and PeriFT was significantly lower than group 2 at the 1st and 2nd months of the surgery (*P* < 0.05 for all variables).

**Conclusions:**

Topical ketorolac combined with steroids is highly efficacious in order to prevent increment in thickness on each part of the macula even after an uneventful phacoemulsification surgery comparing to steroid monotheraphy.

## 1. Introduction

Cystoid macular edema (CME) is the most common cause of decreased visual acuity after uneventful cataract surgery. The pathogenesis of CME involves surgical trauma to the ocular tissues that triggers a cascade of inflammatory events resulting in disruption of the blood-retina and/or blood-aqueous barriers [1–3]. Histopathological studies have also supported the role of inflammation in the pathophysiology of both cystoid and diffuse macular edema (ME) developing after cataract surgery [4, 5]. Different modalities have been used to prevent development of ME by suppressing the inflammation. Topical corticosteroids given during the postoperative period are drugs used most commonly for this purpose. Topical nonsteroidal anti-inflammatory drugs (NSAIDs) are another class of drugs for the management of ME. Several studies have demonstrated the efficacy of NSAIDs in both prevention and treatment of postsurgical ME [6–9]. Optical coherence tomography (OCT) facilitates objective quantification of cystoid and diffuse ME by measuring the volume of the retina. Unlike fluorescein angiography (FA), OCT is a noninvasive method that evaluates different retinal layers and measures multiple parameters associated with ME. In addition, OCT has a high resolution and a higher sensitivity than FA. Several studies reported that ME due to cataract surgery can be detected with OCT when it is not detectable by clinical examination or FA [10, 11]. The purpose of the present study was to objectively evaluate the effects of topical 0.5% ketorolac in combination with topical steroids on macular thickness changes, in cases that had uneventful phacoemulsification and intraocular lens (IOL) implantation, measured by the Fourier domain OCT.

## 2. Materials and Methods

This study was approved by the Ethics Committee of Ataturk Training and Research Hospital. Written informed consent was obtained from each case, and the tenets of the Declaration of Helsinki were followed throughout the study.

This prospective study included 58 eyes of 58 consecutive cases undergoing uneventful phacoemulsification and IOL implantation. Using a randomly generated list of identification numbers, cases were randomly assigned in a 1 : 1 ratio to the ketorolac/steroid group (group 1, *n* = 29) and to the steroid group (group 2, *n* = 29). A complete ophthalmic examination including visual acuity testing, anterior segment examination, intraocular pressure measurement, dilated fundus examination, and macular OCT assessment was performed for all cases at baseline and at 1 week, 1 month, and 2 months after surgery with the same OCT device.

Optical coherence tomography examinations for macular assessment were performed by an experienced retina specialist (YT) using the Fourier domain OCT (RTVue-100, software, version 3.0; Optovue, Fremont, CA, USA). The RTVue-100 has a 5 *μ*m axial resolution and a scan rate of 26,000 axial scans per second. All measurements were performed using macular mapping 5 (MM5) (5 × 5 mm^2^ grid of 11 horizontal and 11 vertical lines with 668 A-scans each and an inner 3 × 3 mm^2^ grid of six horizontal and six vertical lines with 400 A-scans each) and three-dimensional (3D) macula (128 line raster with 512 A-scans each, within 6 × 6 mm2) protocols. Scans were checked to be artifact-free, to go through the center of the full fovea, to be centered on the screen, and to have high signal strength. The MM5 was divided into nine Early Treatment of Diabetic Retinopathy Study (ETDRS) subfields with the central area defined as a 1 mm diameter circle centered on the fovea. The other areas consisted of an inner ring (parafovea) and an outer ring (perifovea). The parafoveal and perifoveal rings were divided into four sectors; the superior, nasal, temporal, and inferior sectors.

To evaluate anatomical changes in the macular area during the follow-up period, each measurement points were selected exactly on the same location. With the help of eye-tracking system and internal fixation target, exact localizations were achieved in every evaluation period. Images with a signal strength index (SSI) of 40 or greater were used for analysis.

The mean foveal thickness (MFT; central 1 mm diameter centered on the fovea), parafoveal thickness (ParaFT; central 3 mm diameter centered on the fovea), and perifoveal thickness (PeriFT; central 5 mm diameter centered on the fovea) were automatically calculated by the mapping software.

The presence of any liquid or cystoid changes was also assessed. Eyes that developed CME were excluded from the statistical analyses.

Cases with a history of intraocular surgery, uveitis, glaucoma, macular pathology including posterior vitreous detachment with or without vitreomacular traction or other ocular diseases, and having diabetes were not included in the study. Eyes with posterior subcapsular/polar cataracts or any other condition that precluded successful OCT examination; eyes with any surgical complications such as intraoperative posterior capsule rupture, vitreous loss, or iris damage; and eyes needing any additional intracameral injections with agents such as adrenaline were also excluded.

All surgeries were performed by the same surgeon (YT). Phacoemulsification was performed under local anesthesia with the same technique using the same materials such as viscoelastic substance and IOL in all eyes. After clear corneal tunnel incisions and sodium chondroitin sulfate-sodium hyaluronate (Viscoat®, Alcon, Fort Worth, TX, USA) injections into the anterior chamber, capsulorhexis and hydrodissection were performed. The entire nucleus was removed with ultrasonic power, and the cortex was completely removed by aspiration. Same balanced salt solution (BSS Plus® Alcon Laboratories, Texas, USA) was used as an irrigation solution in each operation without any additional drug including mydriatics and analgesic. Following the injection of sodium hyaluronate (Bio-Hyalur Plus®, Bio-tech, New Delhi, India), foldable hydrophilic acrylic IOLs of the same brand (OcuFlex®, Polymer Technologies, Padra, India) were implanted into the capsular bag in each eye. Viscoelastic materials were carefully removed from the anterior chamber and the inside of the bag after the IOL implantation, and the clear corneal incisions were closed with hydration. Subconjunctival antibiotic and steroid combination was injected. FDA-approved cefuroxime was not available in our hospital. To prevent potential corneal and macular side effects of hospital-prepared antibiotic, we did not inject any intracameral antibiotic. The effective phacoemulsification time (EPT) and the phacoemulsification energy level were noted. All cases received topical lomefloxacin (Okacin®, Novartis, Taby, Sweden) q.i.d. postoperatively for 2 weeks, and due to increased incidence of postoperative iritis in dark color irises, 0.1% topical dexamethasone (Maxidex®, Alcon-Couvreur, Puurs, Belgium) was given seven times daily for 1 week and dexamethasone was tapered slowly and discontinued within 6 weeks in each patient included in the presented study. Cases in group 1 were additionally given topical 0.5% ketorolac (Acular®, Allergan, Mayo, Ireland) q.i.d., beginning 2 days prior to surgery until 4 weeks after surgery. Follow-up visits were performed postoperatively on day 1, week 1, and months 1 and 2.

All statistical analyses were performed using SPSS, for Windows software (ver. 16.0 SPSS Inc., Chicago, IL, USA). Continuous variables were compared with the Mann–Whitney *U* test. Data are presented as means ± standard deviation for all cases. A *P* value < 0.05 was considered statistically significant.

## 3. Results

According to the inclusion criteria, 58 eyes of 58 cases were initially included in the study. Thirteen (seven from group 1 and six from group 2) cases were excluded from the study due to intraoperative complications, missing follow-ups, or inappropriate postoperative drug use.

Two eyes from group 2 were also excluded from the statistical analyses because of the development of cystoid macular edema (CME) detected by OCT evaluation, one of them being clinically significant macular edema (CSME). All the macular thickness parameters including MFT, ParaFT, and PeriFT were significantly higher in these two eyes than that in the rest of the study group. To achieve correct statistical evaluation, excessive values of the eyes with CME were excluded. Forty-three cases (22 eyes in group 1 and 21 eyes in group 2) were included in the final analyses.

No one had dry eye, ocular surface disease, or corneal irritation due to preoperative topical ketorolac use.

The demographical characteristics of cases in group 1 and group 2 are summarized in Table 1. The mean EPTs was 10.7 ± 8.8 seconds and 13.5 ± 11.3 seconds in groups 1 and 2, respectively. The difference was not significantly different (*P* = 0.544). The mean phacoemulsification energy level was 17.0 ± 7.8% in group 1 and 15.8 ± 10.1% in group 2, which was also not significantly different (*P* = 0.679).

The mean baseline MFT was 251.8 *μ*m in group 1 and 246.3 *μ*m in group 2 (*P* = 0.266). At baseline, the mean ParaFT was 318.7 *μ*m in group 1 and 315.3 *μ*m and in group 2 (*P* = 0.827). The mean PeriFT was 288 *μ*m preoperatively in group 1 and 287.7 *μ*m in group 2 (*P* = 0.980). As shown in Table 2, the mean baseline MFT, ParaFT, and PeriFT values did not show any difference between groups 1 and 2 (*P* > 0.05 for comparisons).

The mean baseline MFT in group 1 was 251.8 *μ*m. It was 250.7 *μ*m at week 1 (*P* = 0.081), 252.9 *μ*m at month 1 (*P* = 0.553), and 253.3 *μ*m at month 2 (*P* = 0.423). The MFT changes were not significantly different from the baseline values for each follow-up visit. The mean ParaFT was 318.7 *μ*m at baseline, and it increased to 320.3 *μ*m at week 1 (*P* = 0.313). The mean ParaFT was significantly increased to 322.8 *μ*m at month 1 (*P* = 0.009), and it reached to 323.3 *μ*m at month 2, which was also significantly greater than the baseline level in group 1 (*P* = 0.025). The mean preoperative PeriFT was 288 *μ*m. It was 289.4 *μ*m at week 1, but the difference was not statistically significant from the baseline (*P* = 0.276). The mean postoperative PeriFT increased to 292.3 *μ*m at month 1 and to 292.9 *μ*m at month 2. Both values were statistically different than that at the preoperative period (*P* = 0.005 and *P* = 0.018, resp.).

In group 2, the mean baseline MFT was 246.3 *μ*m. It was 248.2 *μ*m at week 1, 259.6 *μ*m at month 1, and 257.9 *μ*m at month 2. The MFT increments in each postoperative measurements were statistically significant from the preoperative level (*P* = 0.048, *P* ≤ 0.001, and *P* ≤ 0.001, resp.). The mean baseline ParaFT was 315.3 *μ*m. It increased to 319.6 *μ*m, 328.3 *μ*m, and 325.8 *μ*m at week 1, month 1, and month 2, respectively. The increments were statistically significant in each follow-up period too (*P* = 0.011, *P* ≤ 0.001, and *P* ≤ 0.001, resp.). The mean preoperative PeriFT was 287.7 *μ*m. It progressively increased during the postoperative period and reached 291.7 *μ*m at week 1, 296.8 *μ*m at month 1, and 297.1 *μ*m at month 2, all of which were significantly greater than the preoperative level (*P* = 0.026, *P* ≤ 0.001, and *P* ≤ 0.001, resp.).

**Figure 1:**
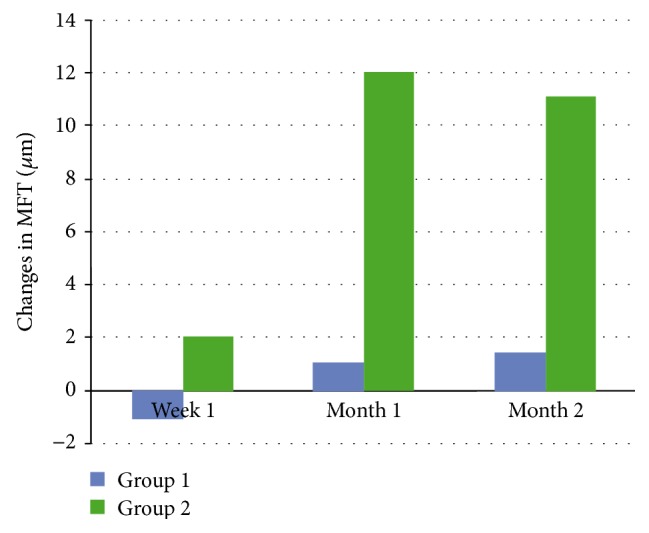
Changes in mean foveal thickness (MFT) in group 1 (ketorolac/steroid group) and group 2 (steroid group). The MFT was significantly lower in group 1 at week 1 and the first and second months postoperatively (*P* = 0.008, *P* ≤ 0.001, and *P* ≤ 0.001, resp.).

Both groups were similar in terms of baseline preoperative macular thicknesses (Table 2). Even though postoperative, each macular thickness parameters were less in group 1 compared with group 2 at week 1, only the difference in the MFT was statistically significant (*P* = 0.008). The mean MFT decreased −1.09 *μ*m in group 1 while it increased 2.06 *μ*m in group 2 at week 1 (Figure 1, Table 3).

Although the increases in ParaFT and PeriFT in group 1 (1.59 ± 5.11 *μ*m and 1.29 ± 4.60 *μ*m, resp.) were lower at week 1 compared with group 2 (2.68 ± 4.11 *μ*m and 2.58 ± 4.90 *μ*m, resp.), the differences were not statistically significant (*P* > 0.05) (Table 4).

During the follow-up period, macular thickness increments were significantly higher in group 2 compared with group 1 for all macular thickness parameters. At postoperative month 1, the mean MFT increment was 12.06 *μ*m for group 2 and only 1.14 *μ*m for group 1 (*P* ≤ 0.001). The mean ParaFT increment was also significantly greater (11.83 *μ*m) for group 2 compared with group 1 (4.09 *μ*m) (*P* = 0.015). The mean PeriFT increment was still significantly greater (9.33 *μ*m) in group 2 compared with group 1 (4.29 *μ*m) (*P* = 0.048).

**Figure 2:**
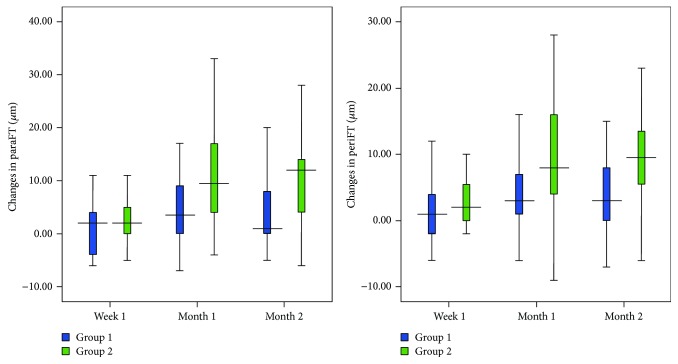
Changes in parafoveal thickness (ParaFT) and perifoveal thickness (PeriFT) in group 1 (ketorolac/steroid group) and group 2 (steroid group). The ParaFT and PeriFT were significantly lower in group 1 at the first and second months postoperatively (ParaFT *P* = 0.015, *P* = 0.015; PeriFT *P* = 0.048, *P* = 0.017, resp.).

At postoperative month 2, the mean MFT increment was significantly greater (11.17 *μ*m) than that of group 1 (1.50 *μ*m) (*P* ≤ 0.001). The mean ParaFT increment was 10.20 *μ*m, while it was 4.55 *μ*m for group 1 (*P* = 0.015). The mean PeriFT increment was also significantly greater (9.15 *μ*m) for group 2 compared with group 1 (4.43 *μ*m) (*P* = 0.017) (Figure 2, Tables 3 and 4).

## 4. Discussion

The present study aimed to assess macular thickness changes under topical ketorolac treatment combination with the routine topical steroid regimen following uneventful phacoemulsification surgery. The results showed that increases in foveal, parafoveal, and perifoveal thicknesses were less pronounced in the eyes that received topical ketorolac in combination with topical steroids.

Despite advances in modern cataract surgery, ME remains one of the major causes of decreased postoperative visual acuity, even after uneventful cataract surgery [12]. Although various hypotheses have been proposed for the pathogenesis of both ME and CME, most clinicians consider anterior segment inflammation as the main causative factor with respect to fluid accumulation within the macula and the development of other complications [3, 7, 13].

Increased prostaglandins and other inflammatory factors in the aqueous humor, which penetrate the vitreous, disrupt the blood-retinal barrier and can cause fluid accumulation in the extracellular space [3, 13]. The leakage of the intravascular contents from dilated perifoveal capillaries initially causes macular thickening, which may progress to cystoid expansions within the outer plexiform layer and inner nuclear layer of the retina [14]. Thickening of the retina is an ongoing process for the development of macular edema, and even small increases may be indicative of pathological changes [10].

In most CME cases, a diagnosis based on clinical findings is impossible [12, 13]. In the evaluation of any stage of ME, OCT is a valuable diagnostic tool that is noninvasive, safe, and repeatable. It provides both qualitative and quantitative valuations of the macula and detects subtle changes that may be overlooked clinically.

Optical coherence tomography can measure different sizes of central macular areas with high reproducibility. Massin et al. reported that the reproducibility of OCT in measuring retinal thickness was within ±5% in normal subjects [15]. Depending on the clinical study, the “foveal thickness” is sometimes central foveal thickness, minimal foveal thickness, or mean foveal thickness (MFT). It has been reported that for assessing changes in macular thickness, the MFT value is more reliable than the minimal foveal thickness, and the repeatability of MFT is better than the minimal foveal thickness [11, 16, 17]. In the present study, we therefore evaluated the MFT and by measuring the average thickness of the area within a 1 mm diameter of the fovea.

The results of the present study show that thickening of the MFT was significantly lower in eyes that received combined ketorolac and topical steroid than in eyes receiving only topical steroid at each postoperative observation period. The mean increase in ParaFT and PeriFT was similarly significantly lower in eyes that received combined ketorolac and topical steroid treatment compared with eyes that received only steroid drops postoperatively at months 1 and 2.

Numerous studies, based on OCT evaluating macular thickness changes, which compared either with the contralateral nonoperated eye or the preoperative values of the operated eye, have reported that macular thickness increases in eyes even after uneventful cataract surgery [13, 18–20]. The macular thickness generally reaches its peak value about 6 weeks after cataract surgery. Even though we did not measure macular thickness exactly at postoperative week 6, the peak macular thickness level was observed during months 1 and 2, which has included postoperative week 6. Consistent with our findings, Perente et al. [18] observed a significant increase in the macular thickness within the first month after an uneventful cataract surgery. In their study, increased macular thicknesses lasted for 6 months, during which time the patients continued to use steroids. However, they did not follow-up the patients after 6 months, and they did not remark on the final status of the macula. Cagini et al. reported that the onset of clinically significant CME is rare after uncomplicated phacoemulsification cataract surgery. They found that when compared with preoperative values, an asymptomatic increase in macular thickness and volume was observed at postop week 12 in eyes receiving only steroids during the first postoperative 6 months.

Both Asano et al. [21] and Miyake et al. [22] observed beneficial effects of topical NSAIDs on macular thickness after cataract surgery. Both these studies reported that less macular thickening was detected in eyes treated with topical NSAIDs compared with eyes treated with only topical steroids. A prospective study by Almeida et al. [23] showed that the addition of topical NSAIDs to a topical steroid regimen resulted in a lower total macular volume at postoperative month 1.

Lee et al. [24] reported that following uncomplicated cataract surgery, topical ketorolac 0.45% was more effective than diclofenac 0.1% in preventing increases in macular thickness and volume. They observed that two months after surgery, the ketorolac group had significantly lower total foveal thickness, total macular thickness, and average macular thickness than the diclofenac group. Additionally, 1 and 2 months after surgery, changes from preoperative values in average macular thickness and total macular volume were significantly less in the ketorolac group than in the diclofenac group.

In the present study, the increases in ParaFT were 11.83 ± 10.29 *μ*m and 10.20 ± 8.59 *μ*m, respectively, at month 1 and month 2 after surgery in eyes that received only topical steroids. The equivalent values were 4.09 ± 6.36 *μ*m and 4.55 ± 8.33 *μ*m, respectively, in eyes that received ketorolac combined with steroids, which was significantly less than that of the steroid only treatment group. The PeriFT increased by 9.33 ± 9.07 *μ*m and 9.15 ± 7.50 *μ*m in the topical steroid group at postoperative month 1 and 2, respectively. The equivalent values were significantly lower in eyes receiving combined treatment (4.29 ± 5.96 *μ*m and 4.43 ± 8.07 *μ*m, resp.). The mean MFT decreased −1.09 *μ*m in the combined treatment group while it increased 2.06 *μ*m in the steroid only treatment group. While the mean MFTs were significantly higher in each postoperative observational period in the steroid treatment group, the MFTs were not significantly different from preoperative levels in the combined ketorolac and steroid treatment group. All these results show that macular thickness increments were significantly prevented by combined topical ketorolac and steroid treatment. The thickness changes were also more prominent in the central fovea in eyes that received only topical steroids; the MFT increased nearly 5% while the increment was <4% both in the ParaFT and PeriFT compared with preoperative values. This represents indirect evidence that topical steroids alone are not sufficient to prevent edema of the central foveal, which is the most sensitive part of the macula that lacks a vascular structure.

To suppress inflammation after cataract surgery, different treatment protocols as well as topical NSAIDs have been used for prophylactic purposes. Roberts [25] reported that the density of anterior chamber flare was significantly lower in eyes that received topical diclofenac for 3 days before the surgery comparing to eyes that were initially treated on day 1 after surgery. The same study also showed that no difference was detected between the group which started diclofenac treatment 1 hour before the surgery and the group that received diclofenac treatment on day 1 after surgery.

Duan et al. [26] performed a meta-analysis study and their results indicated that NSAIDs are effective drugs compared to placebos for the relief of anterior chamber inflammation. They found that diclofenac was the most likely to improve anterior chamber inflammation after cataract surgery, followed by nepafenac, ketorolac, bromfenac, and flurbiprofen.

El-Harazi et al. [27] also reported that no significant difference was detected in inflammatory response between groups given ketorolac 30 minutes before and 1 day after surgery. Bucci and Waterbury [28] studied patients undergoing cataract surgery who received either ketorolac q.i.d. or bromfenac b.i.d. for 2 days preoperatively and reported that ketorolac maintained significantly higher aqueous concentrations and significantly lower aqueous prostaglandin E2 (PGE2) levels than bromfenac. Heier et al. [29] studied the vitreous concentrations of PGE2 in patients treated with different types of topical NSAIDs for 3 days before vitrectomy and reported that vitreous concentrations of PGE2 in patients treated with ketorolac were significantly lower than those who received nepafenac and subjects who did not receive any topical treatment. In the present study, we preferred to start the ketorolac treatment 2 days before surgery to allow the ketorolac to increase aqueous humor saturation levels and to achieve the maximum anti-inflammatory activity during the surgery.

Even though all eyes in each group had similar preoperative characteristics without any preoperative risk factors, with similar phacoemulsification parameters, and without any intraoperative complications or inappropriate drug use, CME development was observed in 2 of 45 (4.4%) eyes at postoperative month 1. Both eyes were in the only topical steroid treatment group. This was indirect evidence of the anti-inflammatory activity of ketorolac and its preventive effect on CME development. In one eye, cystoid changes were not noticed during the fundus examination, but retinal thickening and cystoid changes were detected using OCT. No placebo was used in the control group. This was the only limitation of our study. But overall, topical steroids combined with ketorolac treatment were more effective in the prevention of both clinical and subclinical macular edema than topical steroids alone. As far as we know, our study was the first one that showed the antiedematous effects of NSAIDs that was more prominent in the avascular region, the central fovea, comparing to the vascular parts of the macula. Our study showed the antiedematous effect of both pre- and postoperative use of topical NSAIDs, particularly the use of ketorolac combined with topical steroids, mainly on the central fovea which is the most important part for the visual acuity in eyes that underwent uneventful cataract surgery.

In conclusion, our results demonstrated the protective role of topical ketorolac treatment against the development of CME. In eyes without predisposing risk factors for CME, the addition of ketorolac to topical steroids also had a statistically significant preventive effect on thickness increments in each part of the macula. Starting topical ketorolac 2 days before the surgery minimized the possibility of inflammation.

## 5. The Ethics/Consent

This prospective study was conducted in compliance with the institutional and government review board regulations, informed consent regulations, and the Declaration of Helsinki. Written informed consent was obtained from all patients and the control subjects before the clinical evaluation. This study was approved by the ethics committee of Ankara Ataturk Training and Research Hospital (the number is 20090814).

## Figures and Tables

**Table 1:** Demographical data of patients.

	Group 1 (*n* = 22)	Group 2 (*n* = 21)	*P* value
Male (%)/female (%)	13(59.1%)/9(40.9%)	14(66.7%)/7 (33.3%)	0.402
Age (years)	62.9 ± 10.9	67.4 ± 10.3	0.181
EPT (sec)	10.7 ± 8.8	13.5 ± 11.3	0.544
Phacoenergy (%)	17.0 ± 7.8	15.8 ± 10.1	0.679

Group 1: ketorolac/steroid group; group 2: steroid group; EPT: effective phaco time. (Mann–Whitney *U* test, *P* < 0.05).

**Table 2:** Preoperative macular thickness measurements in groups 1 and 2.

	Group	*N*	Min	Max	Mean	SD	*P* value
MFT (*μ*m)	1	22	226	275	251.8	16.8	0.266
	2	21	224	273	246.3	13.8	
ParaFT (*μ*m)	1	22	290	346	318.7	15.6	0.827
	2	21	259	338	315.3	18.9	
PeriFT (*μ*m)	1	21	252	314	288.0	15.2	0.980
	2	21	246	315	287.7	16.7	

Group 1: ketorolac/steroid group; group 2: steroid group; Min: minimum; Max: maximum; SD: standard deviation; MFT: mean foveal thickness; ParaFT: parafoveal thickness; PeriFT: perifoveal thickness (Mann–Whitney *U* test, *P* < 0.05).

**Table 3:** Postoperative changes in mean foveal thickness.

	Postoperative	Group	*N*	Min	Max	Mean	SD	*P* value
MFT (*μ*m)	1st week	1	22	−6	4	−1.09	2.78	0.008
		2	21	−3	10	2.06	3.65	
	1st month	1	22	−5	13	1.14	4.94	0.000
		2	21	−6	29	12.06	8.47	
	2nd month	1	22	−10	19	1.50	6.62	0.000
		2	21	−1	33	11.17	7.60	

Group 1: ketorolac/steroid group; group 2: steroid group; Min: minimum; Max: maximum; SD: standard deviation; MFT: mean foveal thickness (Mann–Whitney *U* test, *P* < 0.05).

**Table 4:** Postoperative changes in parafoveal thickness and perifoveal thickness.

	Postoperative	Group	*N*	Min	Max	Mean	SD	*P* value
ParaFT (*μ*m)	1st week	1	22	−6	11	1.59	5.11	0.470
		2	21	−5	11	2.68	4.11	
	1st month	1	22	−7	17	4.09	6.36	0.015
		2	21	−4	33	11.83	10.29	
	2nd month	1	22	−5	28	4.55	8.33	0.015
		2	21	−6	28	10.20	8.59	

PeriFT (*μ*m)	1st week	1	22	−6	12	1.29	4.60	0.243
		2	21	−10	10	2.58	4.90	
	1st month	1	22	−6	16	4.29	5.96	0.048
		2	21	−9	28	9.33	9.07	
	2nd month	1	22	−7	29	4.43	8.07	0.017
		2	21	−8	23	9.15	7.50	

Group 1: ketorolac/steroid group; group 2: steroid group; Min: minimum; Max: maximum; SD: standard deviation; ParaFT: parafoveal thickness; PeriFT: perifoveal thickness (Mann–Whitney *U* test, *P* < 0.05).

## References

[1] Sivaprasad S., Bunce C., Wormald R. (2005). Non-steroidal anti-inflammatory agents for cystoid macular oedema following cataract surgery: a systematic review. *British Journal of Ophthalmology*.

[2] Gulkilik G., Kocabora S., Taskapili M., Engin G. (2006). Cystoid macular edema after phacoemulsification: risk factors and effect on visual acuity. *Canadian Journal of Ophthalmology*.

[3] Miyake K., Ibaraki N. (2002). Prostaglandins and cystoid macular edema. *Survey of Ophthalmology*.

[4] Wolter J. R. (1981). The histopathology of cystoid macular edema. *Albrecht von Graefes Archiv für klinische und experimentelle Ophthalmologie*.

[5] Mc Donnell P. J., de la Cruz Z. C., Green W. R. (1986). Vitreous incarceration complicating cataract surgery. A light and electron microscopic study. *Ophthalmology*.

[6] Heier J. S., Topping T. M., Baumann W., Dirks M. S., Chern S. (2000). Ketorolac versus prednisolone versus combination therapy in the treatment of acute pseudophakic cystoid macular edema. *Ophthalmology*.

[7] Donnenfeld E. D., Perry H. D., Wittpenn J. R., Solomon R., Nattis A., Chou T. (2006). Preoperative ketorolac tromethamine 0.4% in phacoemulsification outcomes: pharmacokinetic-response curve. *Journal of Cataract and Refractive Surgery*.

[8] Sandoval H. P., Fernández de Castro L. E., Vroman D. T., Solomon K. D. (2007). A review of the use of ketorolac tromethamine 0.4% in the treatment of post-surgical inflammation following cataract and refractive surgery. *Clinical Ophthalmology*.

[9] Yilmaz T., Cordero-Coma M., Gallagher M. J. (2012). Ketorolac therapy for the prevention of acute pseudophakic cystoid macular edema: a systematic review. *Eye*.

[10] Brown J. C., Solomon S. D., Bressler S. B., Schachat A. P., DiBernardo C., Bressler N. M. (2004). Detection of diabetic foveal edema: contact lens biomicroscopy compared with optical coherence tomography. *Archives of Ophthalmology*.

[11] Von Jagow B., Ohrloff C., Kohnen T. (2007). Macular thickness after uneventful cataract surgery determined by optical coherence tomography. *Graefe's Archive for Clinical and Experimental Ophthalmology*.

[12] Mentes J., Erakgun T., Afrashi F., Kerci G. (2003). Incidence of cystoid macular edema after uncomplicated phacoemulsification. *Ophthalmologica*.

[13] Lobo C. L., Faria P. M., Soares M. A., Bernardes R. C., Cunha-Vaz J. G. (2004). Macular alterations after small-incision cataract surgery. *Journal of Cataract & Refractive Surgery*.

[14] Flach A. J. (1998). The incidence, pathogenesis and treatment of cystoid macular edema following cataract surgery. *Transactions of the American Ophthalmological Society*.

[15] Massin P., Vicaut E., Haouchine B., Erginay A., Paques M., Gaudric A. (2001). Reproducibility of retinal mapping using optical coherence tomography. *Archives of Ophthalmology*.

[16] Chan A., Duker J. S., Ko T. H., Fujimoto J. G., Schuman J. S. (2006). Normal macular thickness measurements in healthy eyes using stratus optical coherence tomography. *Archives of Ophthalmology*.

[17] Paunescu L. A., Schuman J. S., Price L. L. (2004). Reproducibility of nerve fiber thickness, macular thickness, and optic nerve head measurements using StratusOCT. *Investigative Ophthalmology & Visual Science*.

[18] Perente İ., Utine C. A., Öztürker C. (2007). Evaluation of macular changes after uncomplicated phacoemulsification surgery by optical coherence tomography. *Current Eye Research*.

[19] Cagini C., Fiore T., Iaccheri B., Piccinelli F., Ricci M. A., Fruttini D. (2009). Macular thickness measured by optical coherence tomography in a healthy population before and after uncomplicated cataract phacoemulsification surgery. *Current Eye Research*.

[20] Degenring R. F., Vey S., Kamppeter B., Budde W. M., Jonas J. B., Sauder G. (2007). Effect of uncomplicated phacoemulsification on the central retina in diabetic and non-diabetic subjects. *Graefe's Archive for Clinical and Experimental Ophthalmology*.

[21] Asano S., Miyake K., Ota I. (2008). Reducing angiographic cystoid macular edema and blood–aqueous barrier disruption after small-incision phacoemulsification and foldable intraocular lens implantation: multicenter prospective randomized comparison of topical diclofenac 0.1% and betamethasone 0.1%. *Journal of Cataract & Refractive Surgery*.

[22] Miyake K., Ota I., Miyake G., Numaga J. (2011). Nepafenac 0.1% versus fluorometholone 0.1% for preventing cystoid macular edema after cataract surgery. *Journal of Cataract and Refractive Surgery*.

[23] Almeida D. R., Johnson D., Hollands H. (2008). Effect of prophylactic nonsteroidal antiinflammatory drugs on cystoid macular edema assessed using optical coherence tomography quantification of total macular volume after cataract surgery. *Journal of Cataract and Refractive Surgery*.

[24] Lee T. H., Choi W., Ji Y. S., Yoon K. C. (2016). Comparison of ketorolac 0.45% versus diclofenac 0.1% for macular thickness and volume after uncomplicated cataract surgery. *Acta Ophthalmologica*.

[25] Roberts C. W. (1996). Pretreatment with topical didofenac sodium to decrease postoperative inflammation. *Ophthalmology*.

[26] Duan P., Liu Y., Li J. (2017). The comparative efficacy and safety of topical non-steroidal anti-inflammatory drugs for the treatment of anterior chamber inflammation after cataract surgery: a systematic review and network meta-analysis. *Graefe's Archive for Clinical and Experimental Ophthalmology*.

[27] El-Harazi S. M., Ruiz R. S., Feldman R. M., Villanueva G., Chuang A. Z. (2000). Efficacy of preoperative versus postoperative ketorolac tromethamine 0.5% in reducing inflammation after cataract surgery. *Journal of Cataract & Refractive Surgery*.

[28] Bucci F. A., Waterbury L. D. (2009). Aqueous prostaglandin E_2_ of cataract patients at trough ketorolac and bromfenac levels after 2 days dosing. *Advances in Therapy*.

[29] Heier J. S., Awh C. C., Busbee B. G. (2009). Vitreous nonsteroidal antiinflammatory drug concentrations and prostaglandin E_2_ levels in vitrectomy patients treated with ketorolac 0.4%, bromfenac 0.09%, and nepafenac 0.1%. *Retina*.

